# HER-2 Altered Early-Stage Non-Small Cell Lung Cancer Had Better Survival than Triple-Negative Disease

**DOI:** 10.3390/jcm15093481

**Published:** 2026-05-01

**Authors:** Tawee Tanvetyanon, Dung-Tsa Chen, Jhanelle E. Gray

**Affiliations:** H. Lee Moffitt Cancer Center and Research Institute, 12902 Magnolia Drive, Tampa, FL 33612, USA; dung-tsa.chen@moffitt.org (D.-T.C.); jhanelle.gray@moffitt.org (J.E.G.)

**Keywords:** lung cancer, HER2, ERBB2, early-stage

## Abstract

**Background**: A unique group of non-small cell lung cancer (NSCLC) is driven by alterations in HER2. Early studies, mostly from advanced NSCLC, suggest that NSCLC with HER2 alterations confers a poor prognosis. However, modern studies are scant. **Methods**: We performed an analysis of a United States-based database on stage I–III NSCLC patients diagnosed during 2019–2024. Cases with complete data on EGFR, ALK, and HER 2 alterations were included. Study cohorts were divided into: EGFR or ALK alterations (EGFR/ALK group), HER2 alterations (HER2 group), or negative for these alterations (triple-negative group). Outcomes of interest were survival. **Results**: Analyses were performed on data from 3486 patients: 515 patients (15%) in the EGFR/ALK group, 173 patients (5%) in the HER2 group, and 2798 patients (80%) in the triple-negative group. Median disease-free survival (DFS) was 41.3 months, 26.6 months, and 21.2 months, respectively. Median overall survival (OS) was 62.5 months, 63.7 months, and 40.1 months, respectively. Multivariable analysis showed that DFS and OS were significantly worse among the triple-negative group than HER2 group: adjusted HRs 1.44 (95% CI: 1.08–1.90, *p* = 0.01) and 1.94 (95% CI: 1.25–3.01, *p* = 0.003), respectively. In the subgroup of patients with HER2 alterations, no significant difference in DFS or OS was found among patients with HER2 mutation, HER2 amplification, or HER2 overexpression in this exploratory analysis. **Conclusions**: When classified by the status of EGFR, ALK and HER2, early-stage NSCLC patients with HER2 alterations had significantly better DFS and OS than those with triple-negative biomarkers.

## 1. Introduction

*HER2* (Human Epidermal Growth Factor Receptor 2), also known as *ERBB2*, is an important oncogene which encodes membrane tyrosine kinase HER2 [1]. A member of the EGFR (Epidermal Growth Factor Receptor) family, HER2 functions closely with EGFR to form signaling partners, promoting cellular growth [2]. In non-small cell lung cancer (NSCLC), alterations in *HER2* may occur in the form of mutation, amplification, or protein overexpression. *HER2* mutation occurs in about 2–4% of NSCLC patients, typically as in-frame insertions of exon 20 located in the region between Y772 and P780 such as Y772_A775dupYVMA and G776delinsVC/LC/VV/IC [3]. *HER2* amplification and HER2 overexpression are more common than *HER2* mutation, with a prevalence estimate varies widely as high as 23% in some studies [2]. HER2 alterations, regardless of mechanism, have the potential to activate common downstream pathway; and available studies, mostly from advanced NSCLC setting, have associated HER2 alterations with poor prognosis [4].

At present, the United States (US) Food and Drug Administration (FDA) has approved *HER2*-directed therapy including zongertinib, sevabertinib, and trastuzumab deruxtecan for *HER2*-mutated, pre-treated, advanced NSCLC [5,6,7]. Trastuzumab deruxtecan is also indicated for patients with HER2 overexpression ≥3+ immunohistochemistry (IHC) test [5]. However, there is still no HER2-targeted therapy for early-stage NSCLC. On the other hand, for early-stage NSCLC harboring ALK or EGFR alterations, adjuvant or consolidation targeted therapies with alectinib or osimertinib are available for eligible patients [8].

Some studies have reported that HER2 alterations may co-exist with *EGFR* mutation or *ALK* rearrangement [9,10]. Since targeted therapy can be possible for this patient population, this may affect the prognosis of early-stage NSCLC patients with HER2 alterations. To date, there remains a paucity of modern study on early-stage NSCLC with HER2 alteration. In this study, we conducted a population-based observational study of non-metastatic NSCLC patients diagnosed in the US. The main objective of this study is to investigate the impact of HER2 alteration on prognosis.

## 2. Methods

### 2.1. Patients and Cohort Selection

This population-based analytic study was conducted based on a US-based Flatiron Health Research Database, including data from over 800 cancer care sites [11]. The database consisted of patients who were diagnosed with stage I–III NSCLC during 2019 to 2024. In this analysis, only patients who had complete data on the status of EGFR, ALK, as well as HER2 were included. Study cohorts were divided into 3 groups: those with HER2 alterations, those with EGFR or ALK alterations, and those without any of these alterations, now called a triple-negative group.

### 2.2. Biomarker Testing and Classification

Data on oncogene alteration was supplied by laboratory providers and available only for EGFR, ALK, HER2, and PD-L1. In this study, HER2 alteration included gene mutation, gene amplification or protein overexpression. Methodology by major laboratory providers was provided (Supplemental Table S1). Mutation was tested by next generation sequencing (NGS) either from blood or tissue specimen. Amplification was tested by NGS or fluorescence in situ hybridization (FISH), identifying average gene copy number ≥ 6 copies per cell, or a gene-to-chromosome ratio based on a ratio between *HER2* and centromere of chromosome-17 ≥ 2. HER2 expression was tested by IHC performed on tissue specimen with overexpression defined as 2+ or 3+ [2]. Patients with a positive test for HER2 were classified into the HER2 group, while those with at least one negative test result for HER2 were classified as not HER2 altered. For analytic purposes, patients with both *HER2* mutation and amplification were analyzed with *HER2* mutation. Those with both amplification and overexpression were analyzed with *HER2* amplification. *EGFR* mutation was defined as activating mutation by NGS or polymerase chain reaction and ALK alteration was defined as rearrangement by FISH or IHC. The triple-negative group consisted of patients who were not HER2 altered and without EGFR or ALK alterations.

### 2.3. Covariate Definition

Staging was based on pathological stage according to American Joint Committee on Cancer in lung cancer, 7th edition. When pathological stage was unavailable, clinical stage was used for analysis. For patients with multiple PD-L1 levels observed, the highest level was used for analysis. Socioeconomic status (SES) was based on data obtained from area level according to Yost index. Region classification was based on the US Census Bureau. Treatment was inclusive of neoadjuvant, adjuvant and definitive chemoradiation in early-stage setting. Treatment administered in the setting of advanced disease was excluded. Chemotherapy was identified via utilization of cisplatin or carboplatin. Immunotherapy was identified via utilization of PD-(L)1 checkpoint inhibitors. Radiotherapy technique was classified as external beam or stereotactic treatment.

### 2.4. Outcome Measurement

Outcomes of interest were real-world disease-free survival (DFS) and overall survival (OS). DFS was calculated from the date of diagnosis to the date of progressive disease or death whichever occurred first. OS was calculated from the date of diagnosis to the date of death. For censored cases, the last clinic visit date was used to calculate survival time. To protect confidentiality, the date of death was not available and the 15th of the month, a statistically neutral date, was used for survival estimation.

### 2.5. Statistical Analysis

Descriptive statistics including median for scale variables and proportion for categorical variables were used. Pearson’s Chi-square was used to compare categorical variables. DFS and OS were estimated based on the Kaplan–Meier method. Log rank test was used to compare time to event outcomes. Multivariable analysis of factors associated with DFS or OS were performed using Cox proportional hazards model. Independent variable selection was conducted using a backward, stepwise method with significant level to stay set at 0.05. Assessment of potential effect modifications was conducted by adding interaction terms into the statistical models. All *p*-values were two tailed and significant level was set at <0.05. All analyses and graphics were performed on SPSS version 31.0, IBM, Armonk, NY, USA.

## 3. Results

### 3.1. Patient Characteristics

**Figure 1:**
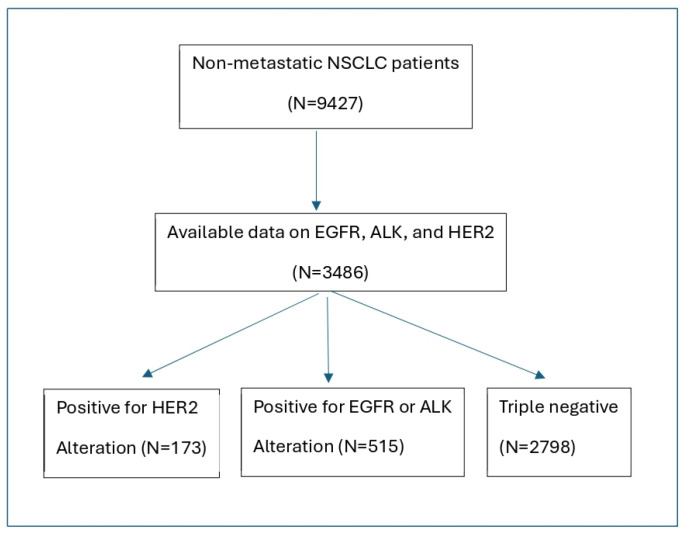
Study cohort identification.

Among 9427 patients with non-metastatic NSCLC in the database, 3486 patients (37%) had complete data on EGFR, ALK and HER2 alterations, forming the basis of this analysis (Figure 1). The characteristics of patients with and without complete data were comparable (Supplemental Table S2), There was a slightly higher proportion of female, adenocarcinoma, and non-smoker patients among those with complete data.

For those with complete biomarker data, the median age was 71 years (range: 35–85 years). Among 3486 patients, 173 patients (5%) were classified into the HER2 group, 515 patients (15%), into the EGFR/ALK group, and 2798 patients (80%), into the triple-negative group (Table 1). Several features were distinct among groups. A higher proportion of adenocarcinoma and never-smoker was observed in HER2 and EGFR/ALK groups. The proportion of female sex and Asian race was highest in the EGFR/ALK group but appeared comparable between the HER2 group and the triple-negative group. Moreover, the EGFR/ALK group also contained the highest proportion of stage I patients. The EGFR/ALK group also underwent more surgery, had more chemotherapy and had less immunotherapy than other groups. No significant difference among groups in age and insurance status was found.

Data from drug utilization file showed that adjuvant or consolidation targeted therapy was used among 139 patients (27%) in the EGFR/ALK group, with osimertinib in 128 patients, alectinib in 8 patients, and afatinib in 2 patients. In the HER2 group, 7 patients (4%) received osimertinib. All of them had co-existing EGFR mutation. In the triple-negative group, 7 patients (0.3%) received targeted therapy, consisting of osimertinib in 5 patients, alectinib in 1 patient, and afatinib in 1 patient. No trastuzumab deruxtecan utilization was observed in this study.

### 3.2. Biomarker Characteristics

Of 3486 patients in this analysis, NGS was performed in 2879 patients (83%). Tissue-based testing occurred in 68% of patients and blood-based testing occurred in 32% of patients, based on the first performed test. Laboratory providers were: Guardant (19%), in-house pathology laboratory (19%), Foundation Medicine (16%), Neo-genomics (11%), Tempus (10%), and Caris Life Science (10%). There were 16 patients with dual gene alterations. These consisted of *EGFR* mutation and *ALK* fusion (1 patient), *EGFR* and *HER2* mutation (3 patients), *EGFR* mutation and HER2 overexpression (12 patients), and *EGFR* mutation and *HER2* amplification (1 patient). The biomarker characteristics of patients in the EGFR/ALK group (N = 515) were the following: *ALK* rearrangement 72 patients (14%), *EGFR* L858R mutation 200 patients (39%), *EGFR* exon 19 deletion 197 patients (38%), *EGFR* exon 20 insertion 39 patients (8%), and *EGFR* T790M with or without other *EGFR* alterations 6 patients (1%).

### 3.3. Disease-Free Survival

**Figure 2:**
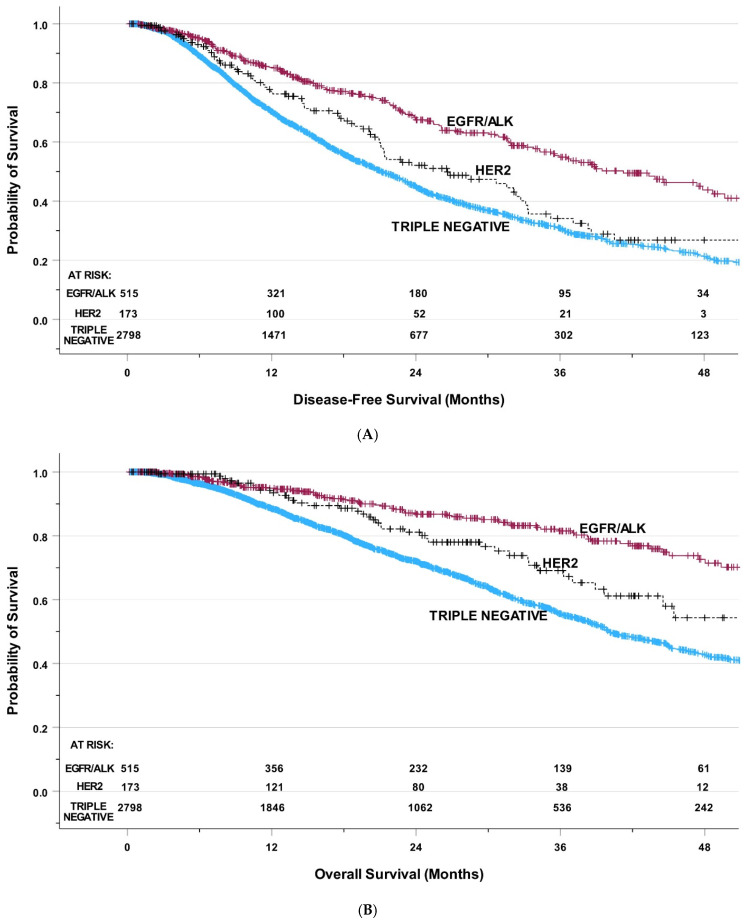
(**A**) Disease-free survival classified by biomarker group. (**B**) Overall survival classified by biomarker group.

At the time of analysis, 1685 patients had developed recurrence or died. The median DFS for all patients was 23.2 months. In the HER2 group, the median DFS was 26.6 months (95% CI: 17.8–35.4 months) (Figure 2A). In the EGFR/ALK group, the median DFS was 41.3 months (95% CI: 34.4–48.1 months). Finally, in the triple-negative group, the median DFS was 21.2 months (95% CI: 19.9–22.4 months). There was a significant difference among groups, *p* < 0.001.

### 3.4. Overall Survival

At the time of analysis, 1019 patients had died. The median OS for all patients was 45.1 months. In the HER2 group, the median OS was 63.7 months (95% CI: 39.9–87.6 months) (Figure 2B). In the EGFR/ALK group, the median OS was 62.5 months (95% CI: 60.0–64.9 months). Lastly, in the triple-negative group, the median OS was 40.1 months (95% CI: 37.7–42.5 months). A significant difference among groups was present, *p* < 0.001.

### 3.5. Multivariable Analysis

We performed a multivariable analysis incorporating patient-related factors (age, sex, race, SES, region, smoking history, performance status), cancer-related factors (biomarker group, histology, stage, PD-L1 level) and treatment-related factors (surgery, external-beam radiotherapy, chemotherapy, immunotherapy). Potential effect modifications were also assessed. Several factors were significantly associated with survival (Table 2). For DFS, biomarker group, surgery, stage, performance status, chemotherapy, sex, region, and immunotherapy were significantly associated with DFS. In addition, the interaction between immunotherapy and biomarker group was statistically significant in that immunotherapy was associated with decreased DFS in the EGFR/ALK group but increased DFS in other groups, *p*-interaction = 0.002. Specifically, among 2971 patients without EGFR/ALK alteration, those who received immunotherapy (N = 895) had a median DFS of 23.4 months compared with 20.7 months among those who did not receive immunotherapy (N = 2076), *p* < 0.001 (Supplemental Figure S1). On the other hand, among 515 patients with EGFR/ALK alteration, those who received immunotherapy (N = 36) had a median DFS of 25.9 months compared with 43.9 months among those who did not (N = 479), *p* = 0.01 (Supplemental Figure S2).

For OS, biomarker group, surgery, histology, stage, performance status, sex, race, and region were identified as significant predictors of OS. After adjusting for other independent variables in the final analytic model, HER2 group had significantly better DFS and OS than the triple-negative group. However, DFS and OS in the EGFR/ALK group appeared numerically the best.

### 3.6. Subgroup Analysis

We performed a subgroup analysis among the HER2 group classified by type of HER2 alterations. The type of HER2 alterations was not mutually exclusive in that among 173 patients with HER2 alterations, 10 patients (6%) were found to have dual types of HER2 alterations. Specifically, 2 patients had mutation plus amplification, analyzed with HER2 mutation group and 8 patients had amplification plus overexpression, analyzed with HER2 amplification group. As a result, this analysis included 57 patients in *HER2* amplification group, 58 patients in *HER2* mutation group, and 58 patients in HER2 overexpression group (Table 3). The characteristics among groups were comparable in terms of age, SES index, performance status and treatment. However, a higher proportion of female patients and stage I disease was observed in the HER2-overexpressed group. Squamous histology was more common in the *HER2*-amplified group, at 26%, compared with 8% in other groups.

**Figure 3:**
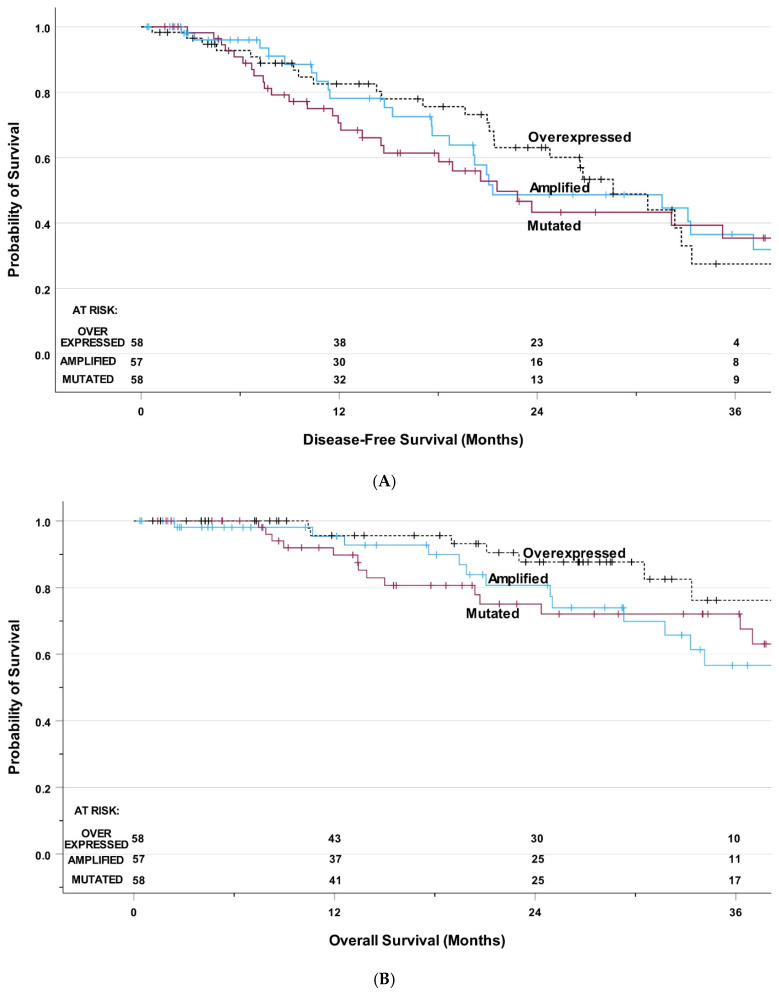
(**A**) Disease-free survival among patients with HER2 alterations. (**B**) Overall survival among patients with HER2 alterations.

DFS and OS were not significantly different by type of HER2 alterations. Specifically, the median DFS was 21.3 months (95% CI: 7.0–35.6 months) for *HER2*-amplified patients, 21.7 months (95% CI: 15.8–27.4 months) for *HER2*-mutated patients, and 28.6 months (95% CI: 23.3–33.9 months) for HER2-overexpressed patients, *p* = 0.66 (Figure 3A). Median OS was not reached for *HER2*-amplified patients, 44.5 months (95% CI: 37.6–51.5 months) for *HER2*-mutated patients, and 64.1 months (95% CI: not estimable) for HER2-overexpressed patients, *p* = 0.07 (Figure 3B). Multivariable analysis incorporating patient-, cancer- and treatment-related variables showed that the type of HER2 alteration was not associated with DFS or OS. For DFS, chemotherapy was the only significant predictor of improved DFS: HR 0.49 (95% CI: 0.28–0.86, *p* = 0.01). For OS, squamous cell carcinoma and lower SES were significant adverse predictors with HR 5.50 (95% CI: 2.07–14.66, *p* < 0.001) and HR 2.62 (95% CI: 1.05–6.49, *p* = 0.04), respectively.

Because *HER2* mutation co-occurred less frequently with *HER2* amplification than did HER2 overexpression, we performed a separate analysis by classifying patients as those with *HER2* mutation (N = 58) vs. those without mutation (N = 115). In this analysis, *HER2* mutation was still not significantly associated with DFS or OS: adjusted HRs 1.41 (95% CI: 0.88–2.26, *p* = 0.15) and 1.73 (95% CI: 0.91–3.27, *p* = 0.09), respectively (Supplemental Figures S3 and S4).

### 3.7. Sensitivity Analysis

In our study there were 16 patients with dual biomarker alterations. Among them, median DFS was 26.7 months (95% CI: 21.2–32.3) and median OS was 45.4 months (95% CI: 43.1–47.7 months). To investigate whether the findings in this study would change if the definition of HER2 group was restricted to include only patients without EGFR alterations, we performed a sensitivity analysis excluding patients with dual EGFR and HER2 alterations. In this analysis, 157 patients were included in the HER2 group.

Within this exclusive HER2 group, median DFS and OS were 26.6 months (95% CI: 17.5–35.7 months) and not reached, respectively. The type of HER2 alterations did not significantly impact DFS: median DFS was 31.6 months (95% CI: 16.4–46.8 months) for *HER2*-amplified patients, 22.8 months (95% CI: 9.7–35.8 months) for *HER2*-mutated patients, and 30.7 months (95% CI: 19.2–42.2 months) for HER2-overexpressed patients, *p* = 0.83. Median OS was not reached for all groups and was not statistically different, *p* = 0.23.

A comparison of outcomes among the exclusive HER2 group, EGFR/ALK group and triple-negative group also yielded similar results as the primary analysis. Specifically, DFS remained worse among the triple-negative group, than the HER2 group (HR 1.37, 95% CI: 1.02–1.84, *p* = 0.04), but better among the EGFR/ALK group than the HER2 group (HR 0.57, 95% CI 0.39–0.83, *p* = 0.003). A statistical interaction between biomarker group and immunotherapy was significant, *p*-interaction = 0.002. In addition, OS remained worse among the triple-negative than the HER2 group (HR 1.98, 95% CI: 1.23–3.19, *p* = 0.005), and numerically better among the EGFR/ALK than the HER2 group (HR 0.82, 95% CI 0.46–1.49, *p* = 0.52).

## 4. Discussion

In this study, when early-stage NSCLC patients were classified into three groups based on EGFR, ALK and HER2 status, those with triple-negative biomarkers appeared to have the worst survival. Despite no available HER2-directed therapy in these early-stage NSCLC patients, those with HER2 alterations still had a significantly better DFS and OS than triple-negative patients. Subgroup analysis conducted among patients with HER2 alterations revealed no significant differences in the outcomes by the type of alterations.

To our knowledge, this is one of the largest studies of HER2 alterations conducted on early-stage NSCLC. The finding that survival is superior among the EGFR/ALK group is not surprising and is in line with some recent studies [12,13]. The availability of adjuvant osimertinib or alectinib as well as consolidation osimertinib likely have contributed to the favorable survival among early-stage NSCLC patients with EGFR/ALK alterations [14,15,16]. On the other hand, in the HER2 group, there is no HER2-directed adjuvant or consolidation therapy available. Nevertheless, DFS and OS among the HER2 group are better than the triple-negative group. This may be due to several reasons.

First, a subgroup of patients with HER2 alterations also carries targetable *EGFR* mutation and may have benefited from available targeted therapy directed against *EGFR* mutation. In this analysis, about 9% of patients in the HER2 group, preferentially among those with HER2 overexpression, also harbored EGFR mutation. Nonetheless, the number of such patients was small and the favorable survival among HER2 group persisted even after excluding these patients from analysis. Another plausible explanation is that HER2 alterations, like *EGFR* mutation, have been linked to minimally invasive adenocarcinoma and this may result in a less aggressive disease biology when compared with the triple-negative group [13]. It has been suggested that lung cancers without driver mutations are biologically more complex and more likely to harbor alterations in tumor suppressor genes such as *TP53*, *KEAP1*, *STK11* or *NF1* [17].

The favorable survival among HER2 group is at odds with some previous studies. For example, a meta-analysis published in 2010 linking HER2 overexpression with poor prognosis [18]. The key differences between that study and the current one are the technology to distinguish patients with EGFR/ALK alterations and the availability of modern chemotherapy as well as immunotherapy. Furthermore, that study included patients with advanced NSCLC. Among advanced NSCLC, studies have shown that the prognosis of patients with HER2 alterations are worse than EGFR/ALK alterations [19]. Some have found *HER2* mutation to be associated with brain metastasis, contributing to poor prognosis in this population [20].

We did not identify the difference in DFS or OS based on the type of HER2 alterations. In line with a previous study conducted in patients with diverse solid tumors, *HER2* mutation is not associated with HER2 overexpression [21,22]. Furthermore, in contrast with breast cancer, *HER2* amplification was only weakly associated with HER2 overexpression in our study. Lastly, the high number of patients with squamous cell carcinoma histology among patients with *HER2* amplification suggests a different disease biology from those with *HER2* mutation. Specifically, some HER2 overexpression may not have been driven by a specific genetic alteration, making them biologically different from HER2 amplification or mutation. Therefore, HER2 alterations likely represent a group of heterogenous diseases, even though in aggregate, they have better survival outcomes than those with triple-negative biomarkers.

While the strengths of this analysis included the database with availability of three types of HER2 alterations, allowing for comparison among groups, there are several constraints. For example, we do not have data on the specific types of *HER2* mutations or gene copy numbers for *HER2* amplification and due to the constraint of population-based study, no central pathology review was possible. We also do not have data on concurrent alterations in other genes beyond *EGFR* and *ALK*. Nonetheless, a previous study on HER2 mutational landscape based on data from Guardant360, one of the major laboratory providers in our study, has been published [9]. We are also limited by the lack of comorbidity data, though this was mitigated by the availability of smoking status, SES, and performance status. Since some patients were prescribed targeted therapy despite the apparent absence of EGFR/ALK alterations, it is possible that we are missing biomarker data or it may have been off-label therapy. However, the number of such patients was small. Finally, the lack of significant survival difference among HER2 variant subgroups may have been due to insufficient sample size and biological differences among the groups. A larger study with a longer follow-up will be necessary to better observe any differences in OS among patients with different types of HER2 alterations.

In summary, when classified based on the status of EGFR, ALK and HER2, patients with early-stage NSCLC and HER2 alterations fared better than those with triple-negative biomarkers. This finding supports more testing for HER2 alterations in this patient population.

## Figures and Tables

**Table 1:** Patient characteristics classified by biomarker group.

Characteristics	HER2 Group N = 173	EGFR/ALK Group N = 515	Triple Negative N = 2798	Total N = 3486	*p*-Value
Sex:					
- Female	94 (54)	360 (70)	1484 (53)	1938 (56)	<0.01
- Male	79 (46)	155 (30)	1314 (47)	1548 (44)	
Age:					
- ≤70 years	91 (53)	245 (48)	1275 (46)	1611 (46)	0.16
- >70 years	82 (47)	270 (52)	1523 (54)	1875 (54)	
Race:					
- White	123 (71)	284 (55)	1930 (69)	2337 (67)	<0.01
- Black	11 (6)	51 (10)	202 (7)	264 (8)	
- Asian	≤5 (≤3)	60 (12)	36 (1)	100 (3)	
- Hispanic	≤5 (≤3)	19 (4)	47 (2)	88 (2)	
- Unknown, mixed	33 (19)	101 (19)	583 (21)	717 (20)	
SES index:					
- Lower	60 (35)	130 (25)	1037 (37)	1227 (35)	<0.01
- Higher	106 (61)	356 (69)	1587 (57)	2049 (59)	
- Unknown	7 (4)	29 (6)	174 (6)	210 (6)	
Payer:					
- Commercial	143 (83)	451 (88)	2340 (84)	2934 (84)	0.23
- Government	24 (14)	51 (10)	376 (13)	451 (13)	
- Others	6 (3)	13 (2)	82 (3)	101 (3)	
Region:					
- Others	63 (37)	184 (36)	1058 (38)	1305 (37)	<0.01
- South	73 (42)	159 (31)	1211 (43)	1443 (41)	
- Unknown	37 (21)	172 (33)	529 (19)	738 (21)	
Smoking history:					
- No	29 (17)	248 (48)	158 (6)	435 (13)	<0.01
- Yes	144 (83)	267 (52)	2640 (94)	3051 (87)	
ECOG:					
- 0	51 (30)	143 (28)	838 (30)	1032 (30)	<0.01
- 1	53 (31)	98 (19)	763 (27)	914 (26)	
- 2	8 (5)	27 (5)	182 (7)	217 (6)	
- unknown	61 (35)	247 (48)	1015 (36)	1323 (38)	
Histology:					
- Non-squamous	146 (84)	505 (98)	1873 (67)	2524 (72)	<0.01
- Not specified	3 (2)	4 (1)	68 (2)	75 (2)	
- Squamous	24 (14)	6 (1)	857 (31)	887 (26)	
Stage:					
- I	70 (41)	286 (55)	1132 (41)	1488 (43)	<0.01
- II	42 (24)	96 (19)	594 (21)	731 (21)	
- III	62 (36)	133 (26)	1072 (38)	1267 (36)	
PD-L1 level:					
- ≤1%	71 (41)	234 (45)	1078 (38)	1383 (40)	0.02
- 1–49%	43 (25)	122 (24)	705 (25)	870 (25)	
- 50–100%	35 (20)	68 (13)	523 (19)	626 (18)	
- Unknown	24 (14)	91 (18)	492 (18)	607 (17)	
Surgery:					
- No surgery	78 (45)	144 (28)	1549 (55)	1771 (51)	<0.01
- Sublobar	14 (8)	63 (12)	266 (10)	343 (10)	
- Lobectomy	75 (43)	294 (57)	933 (33)	1302 (37)	
- Others	6 (4)	14 (3)	50 (2)	70 (2)	
Radiation:					
- No radiation	107 (62)	387 (75)	1455 (52)	1949 (56)	<0.01
- External beam	37 (21)	68 (13)	809 (29)	914 (26)	
- SBRT	26 (15)	53 (10)	450 (16)	529 (15)	
- Unknown type	3 (2)	7 (2)	84 (3)	94 (3)	
Chemotherapy:					
- No	93 (54)	377 (73)	1481 (53)	1951 (56)	<0.01
- Yes	80 (46)	138 (27)	1317 (47)	1535 (44)	
Immunotherapy:					
- No	131 (76)	479 (93)	1945 (69)	2555 (73)	<0.01
- Yes	42 (24)	39 (7)	853 (31)	931 (27)	

Abbreviations: ECOG, performance status according to Eastern Cooperative Oncology Group; SES, socio-economic status; SBRT, stereotactic body radiotherapy.

**Table 2:** Multivariable analysis of factors associated with survival outcomes.

Characteristics	DFS			OS		
	HR	95% CIs	*p*-Value	HR	95% CIs	*p*-Value
Biomarker: - Triple-negative - EGFR/ALK - HER2	1.44 0.60 Reference	1.08–1.90 0.43–0.86	0.01 0.005	1.94 0.94 Reference	1.25–3.01 0.55–1.59	0.003 0.80
Sex: - Female - Male	0.87 Reference	0.77–0.99	0.03	0.79 Reference	0.67–0.94	0.01
Race: - White - Others	NS	NS	NS	0.82 Reference	0.69–0.98	0.03
Region: - South - Others	1.18 Reference	1.04–1.34	0.01	1.29 Reference	1.09–1.52	0.004
ECOG †: - per step increment	1.16	1.06–1.28	0.002	1.57	1.38–1.78	<0.001
Histology: - Squamous - Others	NS	NS	NS	1.25 Reference	1.05–1.50	0.02
Stage: - per step increment	1.41	1.29–1.54	<0.001	1.51 Reference	1.36–1.68	<0.001
Surgery: - Yes - No	0.49 Reference	0.44–0.57	<0.001	0.55 Reference	0.45–0.66	<0.001
Chemotherapy: - Yes - No	0.66 Reference	0.57–0.77	<0.001	NS	NS	NS
Immunotherapy - Yes - No	0.41 Reference	0.35–0.48	<0.001	0.67 Reference	0.55–0.82	<0.001

Abbreviations: DFS, disease-free survival; OS, overall survival; CI, confidence interval; ECOG, performance status according to Eastern Cooperative Oncology Group; NS, variable not significant, unselected in final model † data available from 2163 patients.

**Table 3:** Characteristics of patients with HER2 alteration.

Characteristics	*HER2* Amplified N = 57	*HER2* Mutated N = 58	HER2 Overexpressed N = 58	Total N = 173	*p*-Value
Sex:					
- Female	22 (39)	33 (57)	36 (67)	94 (54)	0.02
- Male	36 (61)	25 (43)	18 (33)	79 (46)	
Age:					
- ≤70 years	33 (58)	27 (47)	31 (53)	91 (53)	0.47
- >70 years	24 (42)	31 (53)	27 (47)	82 (47)	
Race:					
- White	41 (72)	40 (69)	42 (72)	123 (71)	0.76
- Black, Asian, Hispanic	≤5 (≤8)	6 (10)	7 (12)	17 (10)	
- Unknown, mixed	12 (21)	12 (21)	9 (16)	33 (19)	
SES index:					
- Lower	24 (42)	16 (28)	20 (35)	60 (35)	0.36
- Higher	31 (54)	38 (66)	37 (64)	106 (61)	
- Unknown	2 (4)	4 (7)	1 (2)	7 (4)	
Payer:					
- Commercial	47 (83)	50 (86)	46 (79)	143 (83)	0.49
- Government	7 (12)	8 (14)	9 (16)	24 (14)	
- Others	3 (5)	0	3 (5)	6 (4)	
Region:					
- Others	20 (35)	20 (35)	23 (40)	63 (36)	0.004
- South	32 (56)	17 (29)	24 (41)	73 (42)	
- Unknown	5 (9)	21 (36)	11 (19)	37 (21)	
Smoking history:					
- No	6 (11)	12 (21)	11 (19)	29 (17)	0.29
- Yes	51 (89)	46 (79)	47 (81)	144 (83)	
ECOG:					
- 0	14 (25)	19 (33)	18 (31)	51 (30)	0.94
- 1	18 (32)	16 (28)	19 (33)	53 (31)	
- 2	3 (5)	2 (3)	3 (5)	8 (5)	
- unknown	22 (39)	21 (36)	18 (31)	61 (35)	
Histology:					
- Non-squamous	41 (72)	51 (88)	54 (93)	146 (84)	0.01
- Not specified	1 (2)	2 (3)	0	3 (2)	
- Squamous	15 (26)	5 (9)	4 (7)	24 (14)	
Stage:					
- I	20 (35)	21 (36)	29 (50)	70 (41)	0.03
- II	21 (37)	10 (17)	10 (17)	41 (24)	
- III	16 (28)	27 (47)	19 (33)	62 (36)	
PD-L1 level:					
- <1%	22 (39)	28 (48)	21 (36)	71 (41)	0.07
- 1–49%	9 (16)	12 (21)	22 (38)	43 (25)	
- ≥50%	14 (25)	11 (19)	10 (17)	35 (20)	
- Unknown	12 (21)	7 (12)	5 (9)	24 (14)	
Surgery:					
- No surgery	29 (51)	28 (48)	21 (36)	78 (45)	0.51
- Sub-lobar	4 (7)	6 (10)	4 (7)	14 (8)	
- Lobectomy	21 (37)	23 (40)	31 (53)	75 (43)	
- Others	3 (5)	1 (2)	2 (3)	6 (4)	
Radiation:					
- No radiation	31 (54)	36 (62)	40 (69)	107 (62)	0.24
- External beam	15 (26)	15 (26)	7 (12)	37 (21)	
- SBRT	9 (16)	6 (10)	11 (19)	26 (15)	
- Unknown type	2 (4)	1 (2)	0	3 (2)	
Chemotherapy:					
- No	29 (51)	30 (52)	34 (59)	93 (54)	0.66
- Yes	28 (49)	28 (48)	24 (41)	80 (46)	
Immunotherapy:					
- No	43 (75)	41 (71)	47 (81)	131 (76)	0.43
- Yes	14 (25)	17 (29)	11 (19)	42 (24)	

Abbreviations: SBRT, stereotactic body radiotherapy; ECOG, performance status according to Eastern Cooperative Oncology Group; SES, socioeconomic status.

## Data Availability

The data that supported the findings of this study were originated by and are the property of Flatiron Health Inc. Requests for data sharing by license or by permission for the specific purpose of replicating results in this manuscript can be submitted to publicationssataaccess@flatiron.com.

## Supplementary Materials

The following supporting information can be downloaded at: https://www.mdpi.com/article/10.3390/jcm15093481/s1, Figure S1: Disease-free survival among patients with no EGFR/ALK alteration; Figure S2: Disease-free survival among patients with EGFR/ALK alteration; Figure S3: Progression-free survival among patients without co-mutation; Figure S4: Overall survival among patients without co-mutation; Table S1: Methodology of HER2 alteration testing; Table S2: Characteristics of patients with and without complete data on ALK, EGFR, and HER2.
