# Supplementary material for: HER-2 Altered Early-Stage Non-Small Cell Lung Cancer Had Better Survival than Triple-Negative Disease
Source: J Clin Med. 2026 May 1;15(9):3481. doi: 10.3390/jcm15093481 (PMC13164032; doi:10.3390/jcm15093481)
Supplement: Supplementary file 1 [file jcm-15-03481-s001.zip › jcm-4189535-supplementary.pdf]

Supplement Table S1 Methodology of HER2 alteration testing by major laboratory providers as provided online (accessed April 2026)

Laboratory providers	Methodology
**Guardant360**	Guardant liquid biopsy analyzes cell-free DNA from a blood sample, using next-generation sequencing to detect mutations and amplification. The test uses high-throughput, hybridization-based capture technology to detect circulating tumor DNA in the plasma, focusing on HER2 (ERBB2) SNVs (single nucleotide variants), insertions/deletions (indels), and copy number amplifications.
**Foundation Medicine**	Foundation Medicine tests for HER2 (ERBB2) primarily through comprehensive genomic profiling (CGP) using Next-Generation Sequencing to detect gene amplifications and mutations in tissue or blood. They also offer immunohistochemistry (IHC) as an add-on test to evaluate HER2 protein overexpression, using the Ventana PATHWAY HER2 assay. Comprehensive Genomic Profiling (NGS): Tests like FoundationOne®CDx analyze DNA from tumor tissue (FFPE) to identify HER2 amplifications, insertions, deletions, and substitutions. FoundationOne®Liquid CDx analyzes circulating cell-free DNA from blood to identify HER2 alterations when tissue is unavailable. Immunohistochemistry (IHC) is an optional add-on test.
**Neo-genomics**	NeoGenomics tests for HER2 primarily with IHC to detect protein overexpression and Fluorescence In Situ Hybridization (FISH) to detect gene amplification. specifically in breast and gastric cancer. HER2 IHC (Breast/Other) uses antibodies (e.g., Ventana 4B5) to detect the HER2 protein. For breast, IHC results of 3+ are positive; 2+ results are equivocal. FISH is used for confirmation of IHC equivocal (2+) cases or as a direct test to measure HER2 gene amplification relative to chromosome 17 (CEP17).<br>Reflex Testing: If a HER2 IHC test is equivocal (2+), NeoGenomics can reflex the sample to FISH testing for a definitive result.<br>NeoTYPE Profiles: HER2 is included in larger tumor profiles, such as the NeoTYPE Ovarian Tumor Profile, utilizing NGS, FISH, and IHC.
**Tempus**	Tempus tests for HER2 (ERBB2) by combining traditional Immunohistochemistry (IHC) staining with advanced AI-driven analysis of genomic data. IHC tests can be added to next-generation sequencing assay to measure protein expression. Additionally, Tempus uses a proprietary algorithm to analyze RNA-seq data (xR assay) to identify patients likely to benefit from

	further confirmatory HER2 testing. Key aspects of Tempus HER2 testing include: IHC Testing: A specialized staining process (immunohistochemistry) performed on tumor tissue to detect HER2 protein levels, including HER2 low assessments.<br>AI/Machine Learning Prediction: Tempus utilizes a HER2 predictive algorithm (Tempus Next) that analyzes molecular datasets to identify patients with HER2-expressing tumors, flagging them for follow-up testing. Next-Generation Sequencing (NGS): Through its xT and xR assays, Tempus sequences tumor DNA and RNA, which can detect ERBB2 gene amplifications.<br>Reflex Testing: When HER2 is ordered, cases with equivocal results (IHC score 2+) are reflexed to confirmatory ERBB2 FISH (fluorescence in situ hybridization) for confirmation.
**Caris Life Sciences**	Caris Life Sciences tests for HER2 (human epidermal growth factor receptor 2) using a comprehensive, multi-technology approach to identify protein overexpression and gene amplification. They combine Immunohistochemistry (IHC) to measure protein levels (0 to 3+ scale) with Next-Generation Sequencing (NGS) DNA/RNA and In Situ Hybridization (ISH/FISH) to detect gene-level changes. Caris uses standard IHC to assess HER2 protein expression and reflex to in situ hybridization (ISH/FISH) when samples show ambiguous results, allowing for precise quantification of amplification. Comprehensive Molecular Profiling: The platform offersWhole Exome and Whole Transcriptome Sequencing (DNA/RNA) to identify HER2 alterations alongside thousands of other genes.<br>Liquid Biopsy (Caris Assure): A blood-based test that uses circulating nucleic acids (DNA/RNA) to detect HER2 alterations in tumor cells without a tissue biopsy.

Supplement Table S2 Characteristics of patients with and without complete data on ALK, EGFR, and HER2

Characteristics	Without complete information N=5941 (%)	With complete information N=3486 (%)	Total N=9427 (%)	p-value
**Age:**				
**-≤70 years**	3312 (56)	1875 (54)	5187 (55)	0.07
**->70 years**	2629 (44)	1611 (46)	4240 (45)	
**Sex:**				
**-Female**	3081 (52)	1938 (56)	5019 (53)	<0.001
**-Male**	2860 (48)	1548 (44)	4408 (47)	
**Stage*:**				
**-I**	3372 (60)	1488 (43)	4860 (53)	<0.001
**-II**	979 (17)	731 (21)	1710 (19)	
**-III**	1296 (23)	1267 (36)	2563 (28)	
**Smoking history:**				
**-No**	493 (8)	435 (13)	928 (10)	<0.001
**-Yes**	5448 (92)	3051 (87)	8499 (90)	
**Histology:**				
**-Non-squamous**	3583 (60)	2524 (72)	6107 (65)	<0.001
**-Not specified**	123 (2)	75 (2)	198 (2)	
**-Squamous**	2235 (38)	887 (26)	3122 (33)	
**SES index†:**				
**-Lower**	2210 (40)	1227 (38)	3437 (39)	<0.001
**-Higher**	3307 (60)	2049 (62)	5356 (61)	
**Practice:**				
**-Academic**	1573 (27)	687 (20)	2260 (24)	<0.001
**-Community**	4368 (73)	2799 (80)	7167 (76)	

Abbreviations: SES-socioeconomic status

*Data available from 9133 patients

†Data available from 8793 patients

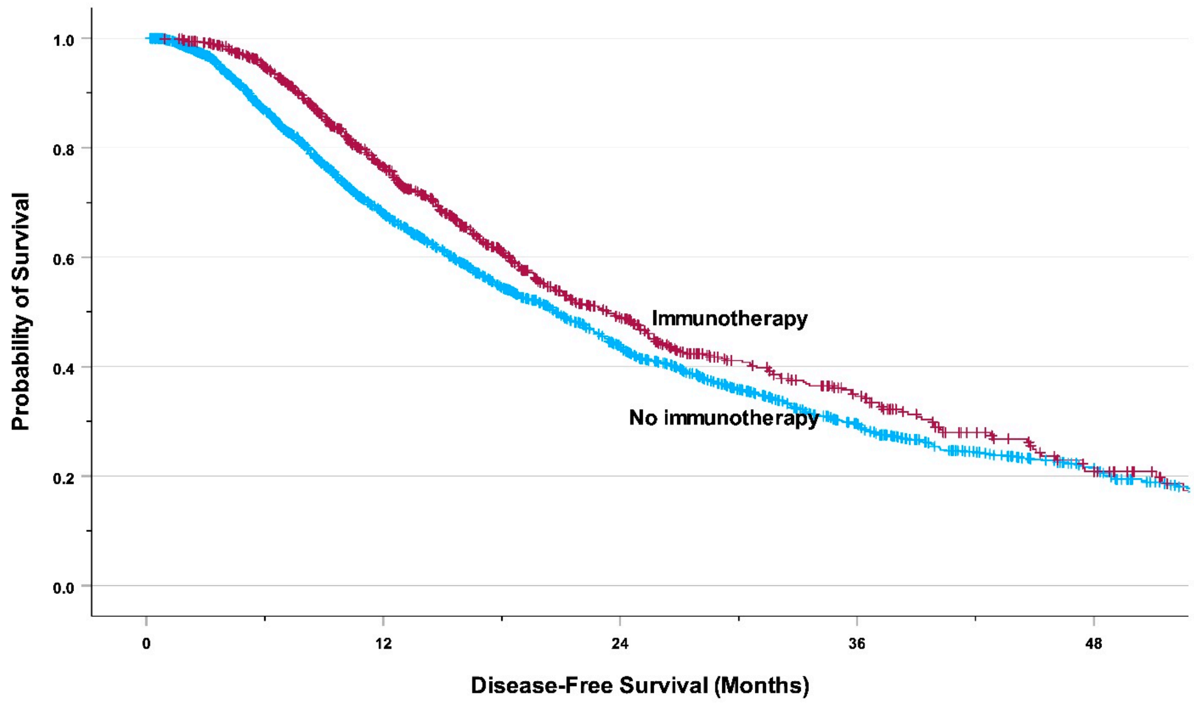

Figure S1 Disease-free survival among patients with no EGFR/ALK alteration

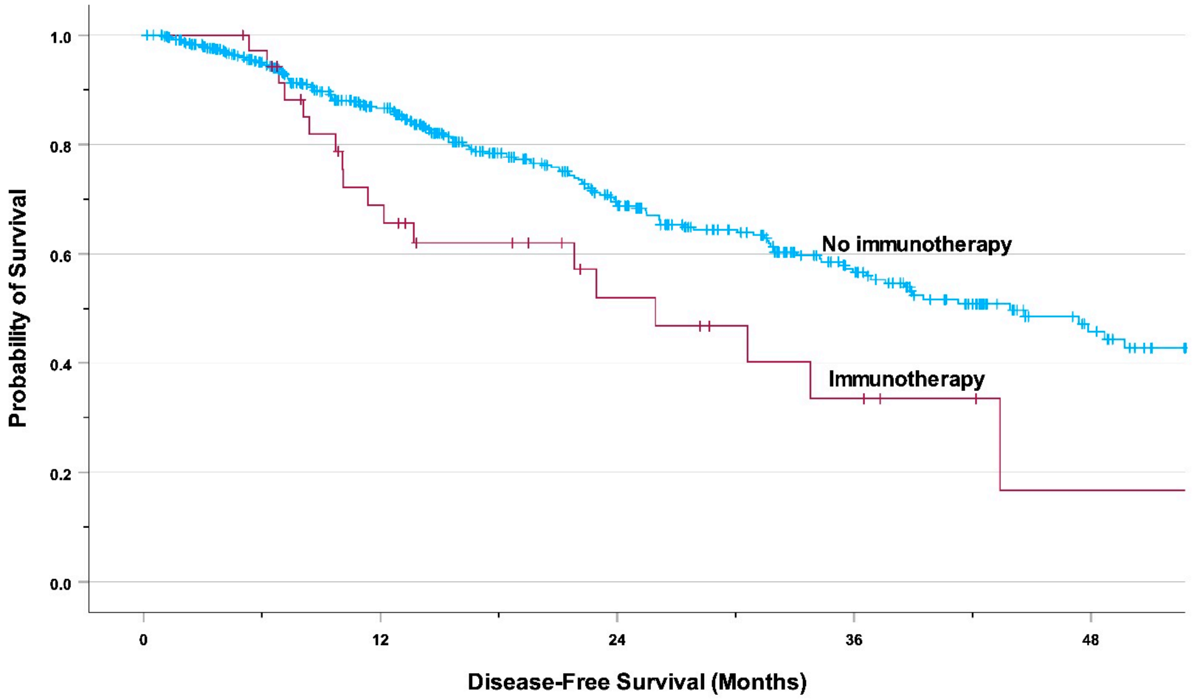

Figure S2 Disease-free survival among patients with EGFR/ALK alteration

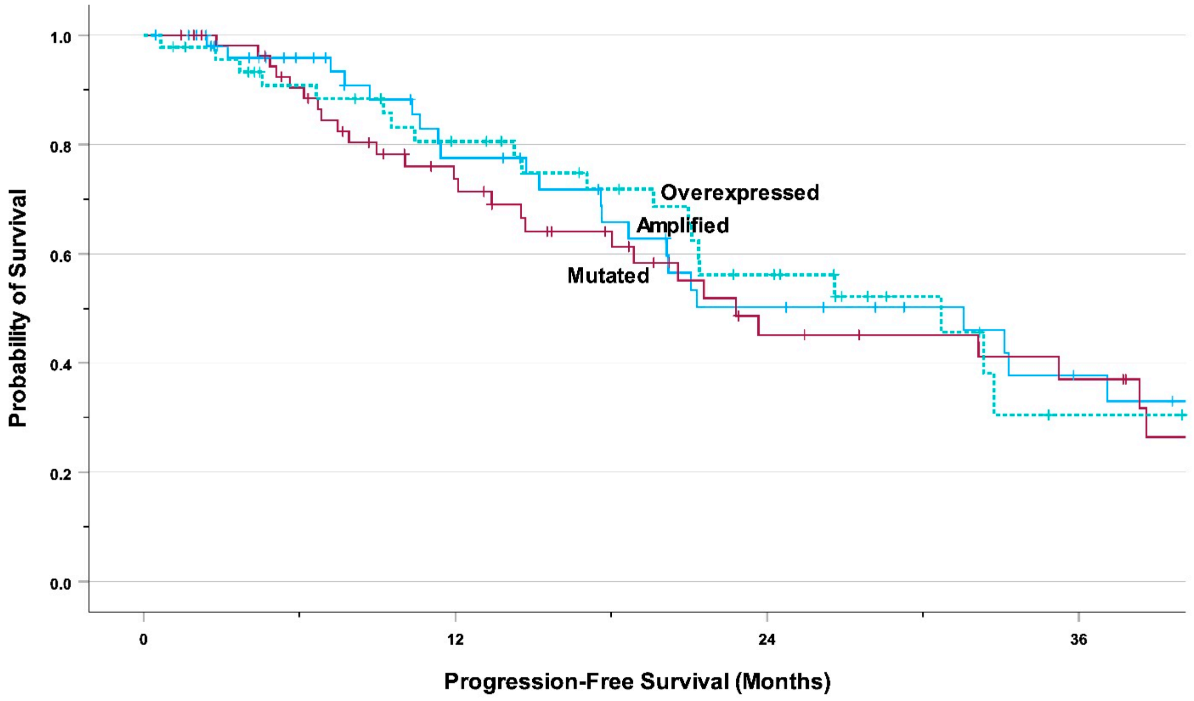

Figure S3 Progression-free survival among patients without co-mutation

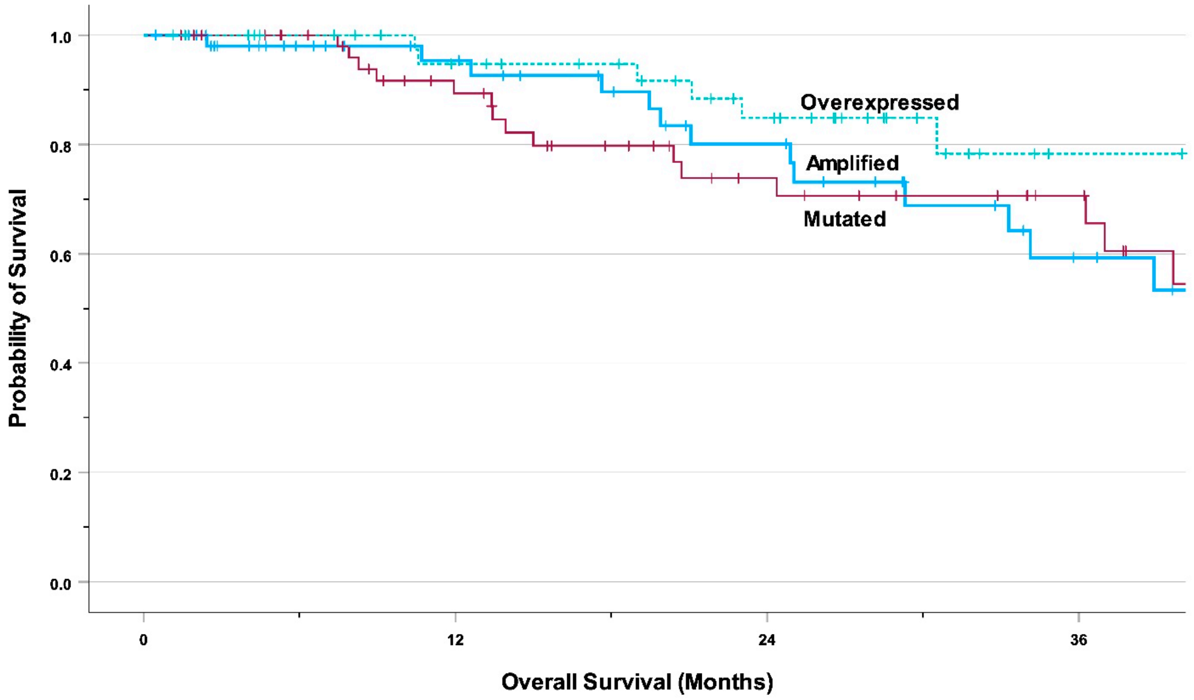

Figure S4 Overall survival among patients without co-mutation
